# Y-27632, a ROCK Inhibitor, Promoted Limbal Epithelial Cell Proliferation and Corneal Wound Healing

**DOI:** 10.1371/journal.pone.0144571

**Published:** 2015-12-16

**Authors:** Chi-Chin Sun, Hsiao-Ting Chiu, Yi-Fang Lin, Kuo-Ying Lee, Jong-Hwei Su Pang

**Affiliations:** 1 Department of Ophthalmology, Chang Gung Memorial Hospital, Keelung, Taiwan; 2 Department of Chinese Medicine, College of Medicine, Chang Gung University, Kwei-shan, Taoyuan, Taiwan; 3 Graduate Institute of Clinical Medical Sciences, College of Medicine, Chang Gung University, Kwei-shan, Taoyuan, Taiwan; Georgetown University, UNITED STATES

## Abstract

Transplantation of ex vivo cultured limbal epithelial cells is proven effective in restoring limbal stem cell deficiency. The present study aimed to investigate the promoting effect of Y-27632 on limbal epithelial cell proliferation. Limbal explants isolated from human donor eyes were expanded three weeks on culture dishes and outgrowth of epithelial cells was subsequently subcultured for in vitro experiments. In the presence of Y-27632, the ex vivo limbal outgrowth was accelerated, particularly the cells with epithelial cell-like morphology. Y-27632 dose-dependently promoted the proliferation of in vitro cultured human limbal epithelial cells as examined by phase contrast microscopy and luminescent cell-viability assay 30 hours after the treatment. The colony forming efficacy determined 7 days after the treatment was enhanced by Y-27632 also in a dose-dependent manner. The number of p63- or Ki67-positive cells was dose-dependently increased in Y-27632-treated cultures as detected by immunofluorescent staining and western blotanalysis. Cell cycle analysis by flow cytometric method revealed an increase in S-phase proliferating cells. The epithelial woundclosure rate was shown to be faster in experimental group received topical treatment withY-27632 than the sham control using a rat corneal wounding model. These resultsdemonstrate that Y-27632 can promote both the ex vivo and in vitro proliferation oflimbal epithelial cell proliferation. The in vivo enhanced epithelial wound healingfurther implies that the Y-27632 may act as a new strategy for treating limbal stem cell deficiency.

## Introduction

The ocular surface is covered by corneal, limbal, and conjunctival epithelial cells that, together with a stable pre-ocular tear film, maintain its integrity. The corneal epithelium exists in a state of dynamic equilibrium, with the superficial epithelial cells being constantly shed into the tear pool. The cells shed from the corneal surface are replaced through proliferation of a distinct subpopulation of cells located at limbal basal layer, known as limbal stem cells (LSCs) [1]. Severe damage to the limbal epithelial cells from various etiologies in the limbal region may lead to loss of the limbal epithelial cells [2], so called limbal stem cell deficiency (LSCD). LSCD, manifested by chronic inflammation, neovascularization, and goblet cell invasion into the cornea, may be complicated by persistent corneal epithelial defects, ulceration, and even perforation of the cornea [3, 4]. The cornea may ultimately be healed by fibrosis, however, the vision will be greatly impaired.

The concept of cell therapy for LSCD is the focus of current research and several innovative therapeutic modalities including limbal transplantation and ex vivo-cultivated limbal stem cells [5, 6] or oral mucosal epithelial cells [7] have been adopted as the surgical procedures in clinical practice. However, rejection issue as well as guarded long term successful rate limited its clinical applications and still waited to be overcome [8, 9]. On the other hand, in patients with partial LSCD, meaning that there are some functionally capable LSCs, simple keratectomy plus amniotic membrane (AM) transplantation seems adequate to prevent further corneal neovascularization [10]. However, structural heterogeneity of AM scaffold limits the therapeutic outcomes for LSCD. Recently, research efforts have focused on designing innovative biocompatible biomaterials with progenitor cells to restore normal ocular surface in patients with LSCD. For example, the hydrogel structure is subjected to modifications which direct stem cell fate [11]. Despite the therapeutic benefits of these biosynthetic materials for LSCD, problems are still remained such as the high material modulus, mechanical interaction with ocular tissue as well as disruption of the pre-ocular tear film [11].

Therefore, pharmacological therapy seems to be a convenient and feasible method to restore impaired limbal stem cell function. Previous studies have demonstrated the effectiveness of Y-27632 (a Rho-associated protein kinase inhibitor, ROCK inhibitor) in regenerating endothelial cells in various animal models with corneal endothelial dysfunction [12, 13]. They found that Y-27632 not only stimulate proliferation, but also reduce apoptosis of corneal endothelial cells [14]. Ras homolog gene family, member A (RhoA) is a small guanosine triphosphatase (GTPase) that functions as a key intracellular regulator of cellular responses including migration and contraction of smooth muscle [15]. Recent study showed that Y-27632 eye drops not only effectively promote corneal endothelial wound healing in a primate animal model, but also improve central corneal edema in patients with endothelial dysfunction [16]. Additionally, inhibition of ROCK has been shown to enhance primate corneal endothelial cell adhesion [13]. However, the role of RhoA/ROCK in limbal epithelial cells has not been examined. Therefore, the present study is designed to identify whether ROCK inhibitor Y-27632 is involved in the regulation of limbal epithelial cell proliferation and cell cycle distribution.

## Materials and Methods

### Materials

Dulbecco’s modified Eagle’s medium (DMEM)/F-12 medium and fetal bovine serum (FBS) were purchased from Invitrogen (Carlsbad, CA, USA). Cell Counting Kit-8 for cell proliferation was purchased from Sigma-Aldrich (St. Louis, Missouri, USA). Y-27632 was from ENZO Life Sciences (Plymouth Meeting, PA, USA). Monoclonal antibodies against Ki67, p63 and K12 were purchased from Thermo Scientific (Fremont, CA, USA), DAKO (Düsseldorf, Germany) and Santa Cruz (Texas, USA), respectively. All studies were performed with the approval of the institutional ethics committee at Chang Gung Memorial Hospital, Taiwan.

### Methods

**Human tissue:** This study has been approved by the authors' Institutional Review Board (103-6066B) at Chang Gung Memorial Hospital, Taiwan. And all clinical investigation has been conducted according to the principles expressed in the Declaration of Helsinki. Corneoscleral buttons isolated from human donor eyes were obtained from the Keelung Chang Gung Memorial Hospital Eye Bank. The written informed consent from the donor for the use of this sample in research was waived by the IRB.

**Animal research:** The Institutional Animal Care and Use Committee (IACUC) at Chang Gung Memorial Hospital, Keelung, Taiwan has approved this study. The use and treatment of rat in this study conformed to the ARVO Statement for the Use of Animals in Ophthalmic and Vision Research.

### Human limbal explants cultured on plastic dishes

The tissue was rinsed three times with DMEM/F-12 containing 50 μg/ml gentamicin and 1.25 μg/ml amphotericin B. After careful removal of excessive sclera, iris, corneal endothelium, conjunctiva, and Tenon’s capsule, the remaining tissue was cut into cubes of approximately 1.5 × 2 × 3 mm^3^ and cultured in a medium made of an equal volume of HEPES-buffered DMEM containing bicarbonate and F-12, and supplemented with 5% FBS, 0.5% dimethyl sulfoxide, 2 ng/ml mouse epidermal growth factor, 5 μg/ml insulin, 5 μg/ml transferrin, 5 ng/ml selenium, 0.5 μg/ml hydrocortisone, 30 ng/ml cholera toxin, 50 μg/ml gentamicin, and 1.25 μg/ml amphotericin B. Cultures were incubated at 37°C under 5% CO_2_ and 95% air, and the medium was changed every 2–3 days, while the extent of each outgrowth was monitored with a phase contrast microscope. After confluence, the expanded limbal epithelial cells were trypsinized and subcultured in 1:4 dilutions. Only the first passage limbal epithelial cells were harvested for subsequent assays.

### Cell proliferation assay

The effects of ROCK inhibitor Y-27632 on limbal epithelial cell proliferation was assayed by the Cell Counting Kit-8 according to the manufacturer’s instructions. In brief, a 96-well plate was pre-coated with bovine collagen solution (Advanced BioMatrix, USA) and pre-incubated in a humidified incubator at 37°C under 5% CO_2_ and 95% air. Cultured limbal epithelial cells were suspended in culture medium and inoculated in a 96-well plate (8 × 10^3^ cells/well) along with various concentrations of ROCK inhibitor Y-27632. After 30 hours incubation, 10 μl of the CCK-8 solution was added to each well of the plate. The plate was incubated for additional 2 hours before measuring the absorbance at 450 nm using a microplate reader.

### Colony forming efficiency assay

Primary expanded limbal epithelial cells were trypsinized, resuspended and inoculated in six-well plates at a seeding density of 1.2 × 10^4^ cells/well. Y-27632 at various concentrations was added into the wells. After seven days of growth of limbal epithelial cells in the described culture conditions, the culture media were aspirated and washed by phosphate-buffered saline (PBS). Total number of cells was photographed and counted after staining with hematoxylin solution (Sigma-Aldrich) for 10 minutes and the colony forming efficiency, i.e. the ability to form colonies (groups of four or more adhering cells derived from the same mother cell), was assessed.

### Immunocytochemical staining

Cultured limbal epithelial cells grown on coverslides or paraffin-fixed corneal tissue sections were fixed and endogenous peroxidase activity was blocked by placing in 2% hydrogen peroxide for 10 min. Slides were rinsed in deionized water and then in Tris-buffered saline containing 0.1% BSA (bovine serum albumin) and 20% normal goat serum for 10 min to block nonspecific staining. Slides were then incubated overnight at 4°C with anti-Ki67, anti-p63 or anti-K12 antibodies at a dilution of 1:100. After thoroughly washing in PBS, the specimens were incubated with fluorescein-conjugated secondary antibody at a dilution of 1:200 for 1 h. The slides were then washed and scanned using a confocal microscope. Negative controls had the primary antibody omitted and DAPI (4',6-diamidino-2-phenylindole) was used for nuclear counterstaining.

### Preparation of cell extracts and Western immunoblot analysis

Cultured limbal epithelial cells grown on coverslides were washed once with PBS and lyzed in the lysis buffer containing 50 mM Tris—HCl, 50 mM β-glycerol phosphate, 50 mM NaCl, 1 mM Na3Vo4, 1 mM EDTA, 1 mM EGTA, 1% NP40, and freshly adding 1 mM DTT, 1 mM PMSF, 2 μg/ml aprodenin, 2 μg/ml leupeptin, and 2 μg/ml pepstatin rightbefore lysis. The cell lysates were then rotated for 30 min and centrifuged at 13,000 × g for 30 min at 4°C and the supernatant was collected. The protein concentration of the cell lysate was determined by using BCA reagent (Thermo Scientific Pierce) with BSA as standards. After adding 1/6 volume of 6× loading dye (50% 2-mercaptoethanol, 1% bromophenol blue), equal amounts of protein (30 μg) from limbal epithelial cells were analyzed by polyacrylamide gel electrophoresis in the presence of sodium dodecylsulfate on a 12% gel (120 V, 2 hours; 4% gel for Ki67) and then transferred onto nitrocellulose membrane, and then incubated with an anti-Ki67 (Thermo Scientific), anti-p63 (DAKO) or anti-Keratin 12 (Santa Cruz) antibodies used at a dilution of 1:1000 in TBST overnight at 4°C. Membranes were washed with TBST four times for 5 minutes each, incubated with a 1:2000 dilution of anti-rabbit, anti-rabbit and anti-goat horseradish peroxidase antibodies for 1 hour, respectively. The membranes were washed with TBST. The immunoreactive bands detected by ECL reagents were developed by Hyperfilm-ECL. Expression of targeted proteins relative to tubulin, lamin B and GAPDH (as the loading controls) was calculated, respectively.

### Analysis of cell cycle distribution and apoptosis by flow cytometry

In vitro cultured human limbal epithelial cells were treated with or without Y-27632 (20μM) at 37°C for 30 hours. Limbal epithelial cells were dissociated to single cells by trypsin digestion, centrifuged at room temperature, fixed in 70% (wt/vol) ethanol, washed, and incubated for 1 hour. After centrifugation at 2000 rpm for 3 minutes, the pellet was resuspended in 500 μl PBS containing 0.5% Triton X-100, 80 μg/ml propidium iodide and 400 μg/ml RNase A and incubated at 37°C for 1 hour. Flow cytometric analyses were then performed using FACSCalibur system (BD Biosciences, Franklin Lakes, NJ).

### In Vivo wound healing assay

Sprague Dawley rats aged 25 weeks and weighed 500 to 600 g, were anesthetized by an intramuscular injection of ketamine (50 mg/kg) and xylazine (10 mg/kg). After a local anesthesia by 0.5% proparacaine hydrochloride eye drops, abrasion of the central 2.5 mm in diameter corneal epithelium was removed from both eyes using corneal rush ring remover (Algerbrush II). After wounding, each cornea was stained with 1% fluorescein, and the area of the epithelial defect was photographed every 24 hours until healed. Measurements of the fluorescein area were computed using image J (Version 1.46, http://rsbweb.nih.gov/ij/index.html). Y-27632 was dissolved in PBS to a concentration of 10 mM and applied (40 μl/drop) topically five times a day (5 AM to 9 PM) to the left eye. The same volume of PBS was applied to the contralateral eye as a control. The topical application of Y-27632 or PBS was continued until complete wound closure was observed. To compare the effects of topical applications, the epithelial healing rate after wounding was calculated for each eye, and the control and treated healing rates were compared by a paired t-test.

### Statistical analysis

Data were analyzed with GraphPad Prism Program (GraphPad, San Diego, http://www.graphpad.com) and expressed as the mean ± SEM and analyzed with a two-tailed Student’s t-test at a p<0.05 level of significance.

## Results

### Y-27632 accelerated the *ex vivo* outgrowth of limbal explants

**Fig 1:**
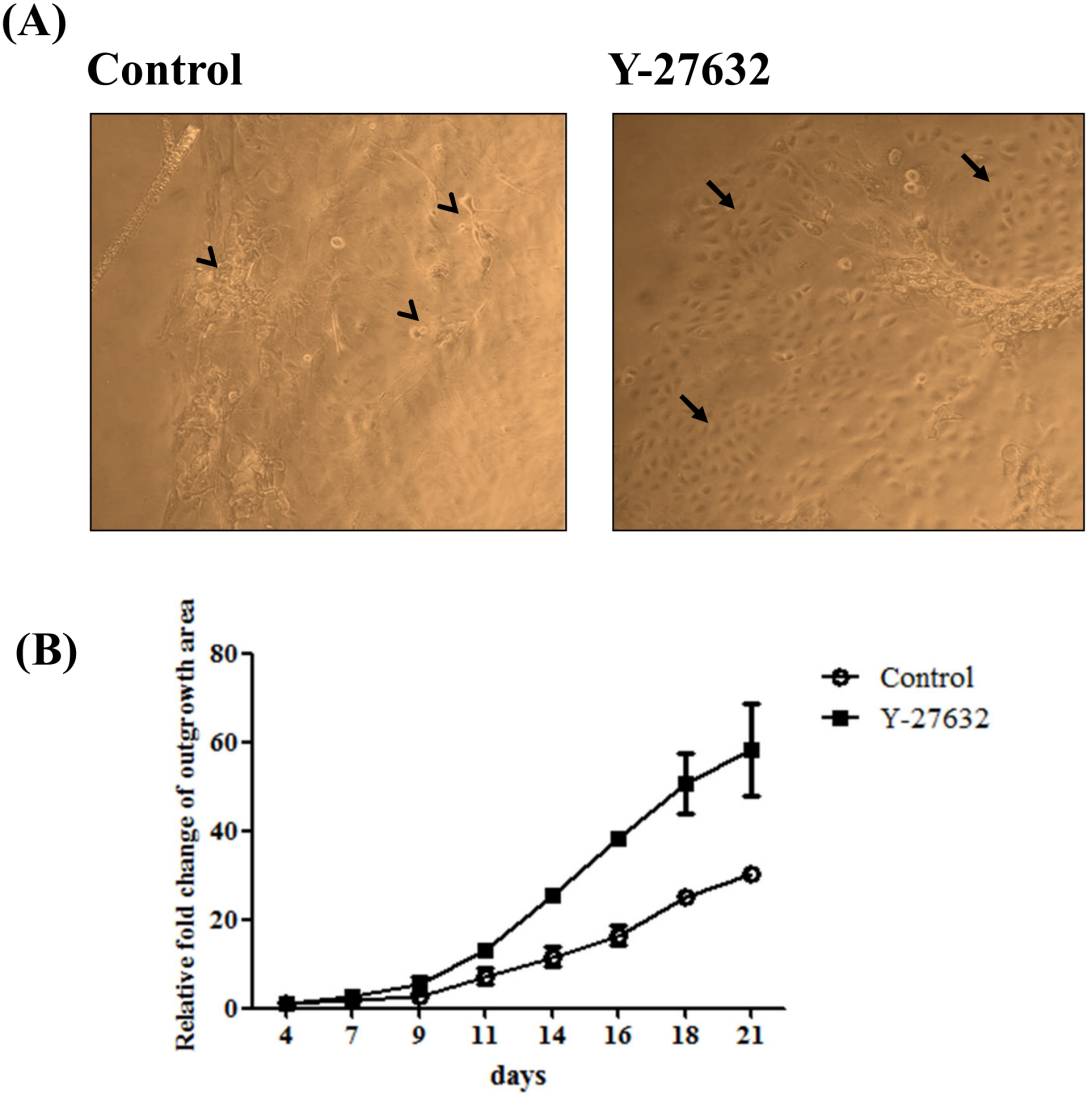
Phase contrast photographs of human limbal explant outgrowth on plastic dishes without or with 10uM Y-27632 at the end of third week in culture. (A). Magnification: 200×. Many pyknotic, fibroblastic-like and irregularly shaped cells were found in cultures without Y-27632 as indicated by arrow heads. A distinct population of cells with prominent nuclei and high nucleus/cytoplasm ratio as indicated by arrows were noted in the presence of 10 μM Y-27632. Quantitative comparison of the outgrowth area between control and Y-27632 group was performed once every two or three days and shown in (B). Statistical analyses were performed using a paired test, and *p<0.05 was considered significant.

To elucidate the effect of Y-27632 on limbal epithelial cell outgrowth, human limbal explants were expanded on plastic dish with or without adding 10 μM Y-27632 for 3 weeks. The appearance and morphological differences between cultures were photographed. Under phase contrast microscopy, many pyknotic, fibroblastic-like or irregular-shaped cells indicated by arrow heads were noted in control group and some were even detaching from the underlying cell layer expanded on plastic dishes (Fig 1A, control). However, in the presence of Y-27632, there were several clusters of expanded limbal epithelial cells with polygonal and cuboidal shape especially in the outer area of explants outgrowth as indicated by arrows (Fig 1A, Y-27632). By measuring and comparing the size of limbal outgrowth area every two or three days, it was clear that Y-27632 significantly accelerated the ex vivo outgrowth rate of limbal explants (Fig 1B).

### Y-27632 promoted the proliferation of expanded limbal epithelial cells in vitro

**Fig 2:**
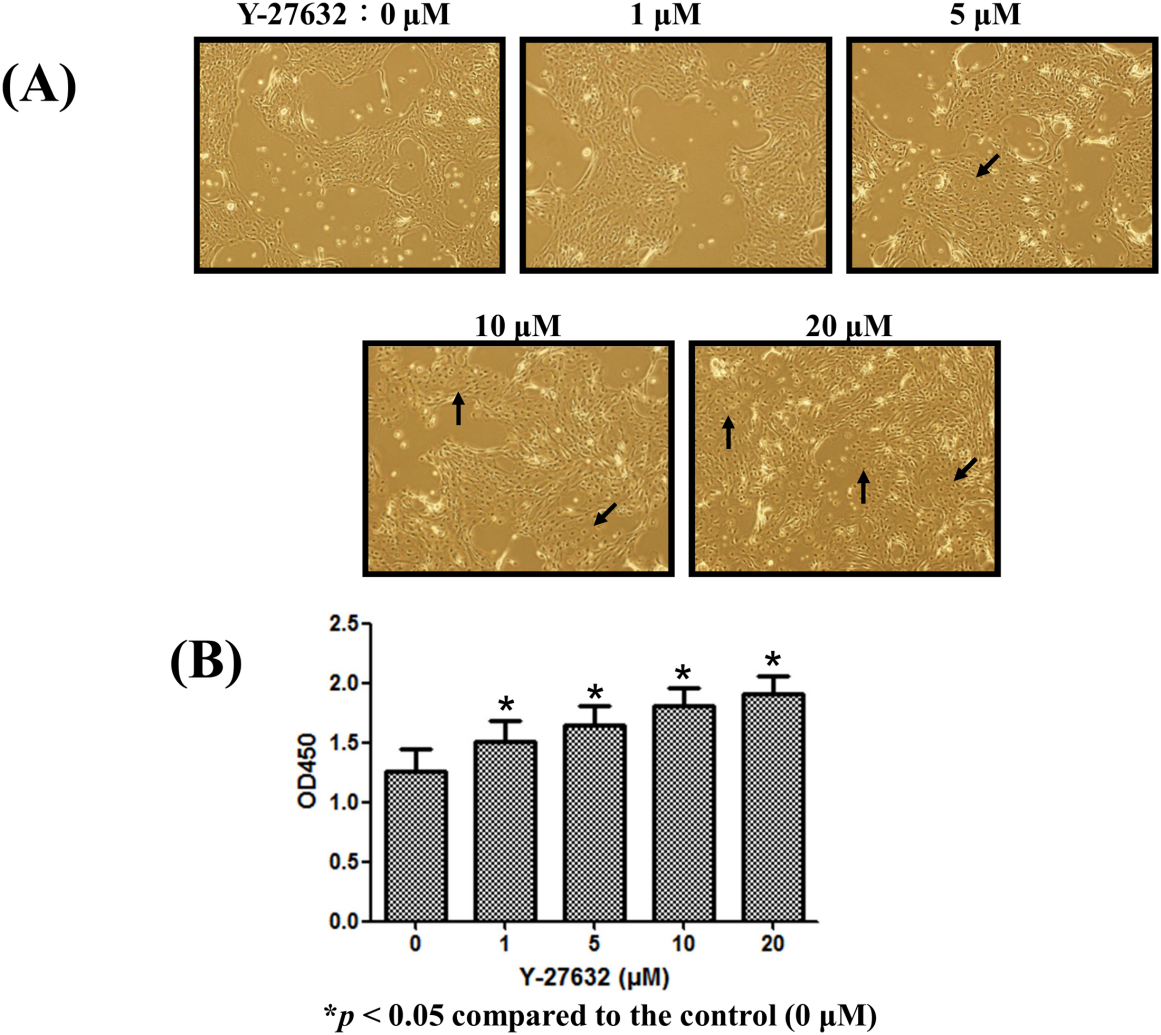
Y-27632 promoted the proliferation of human limbal epithelial cells. Phase contrast photographs of human limbal epithelial cells expanded on plastic dishes with or without adding various concentrations of Y-27632 in culture medium (A). Magnification: 200×. Cells with prominent nuclei and high nucleus/cytoplasm ratio as indicated by arrows were noted in dishes with Y-27632. There was a positive dose-dependent correlation between the concentrations of Y-27632 and proliferation of expanded limbal epithelial cells (B). Statistical analyses were performed using a paired test, and *p<0.05 was considered significant.

To investigate the in vitro effect of Y-27632 on the proliferation of human limbal epithelial cells, various concentrations of Y-27632 were added to cultured human limbal epithelial cells for 30 hours, photographed and assayed by the CCK-8 kit. As shown in Fig 2A, the number of expanded human limbal epithelial cells with prominent nuclei and high nucleus/cytoplasm ratio was increased in a dose-dependent manner by Y-27632. Results from CCK-8 assay revealed that Y-27632 significantly increased the number of in vitro cultured human limbal epithelial cells as compared to the control (p<0.05, Fig 2B).

### Y-27632 enhanced the colony forming efficiency of human limbal epithelial cells

**Fig 3:**
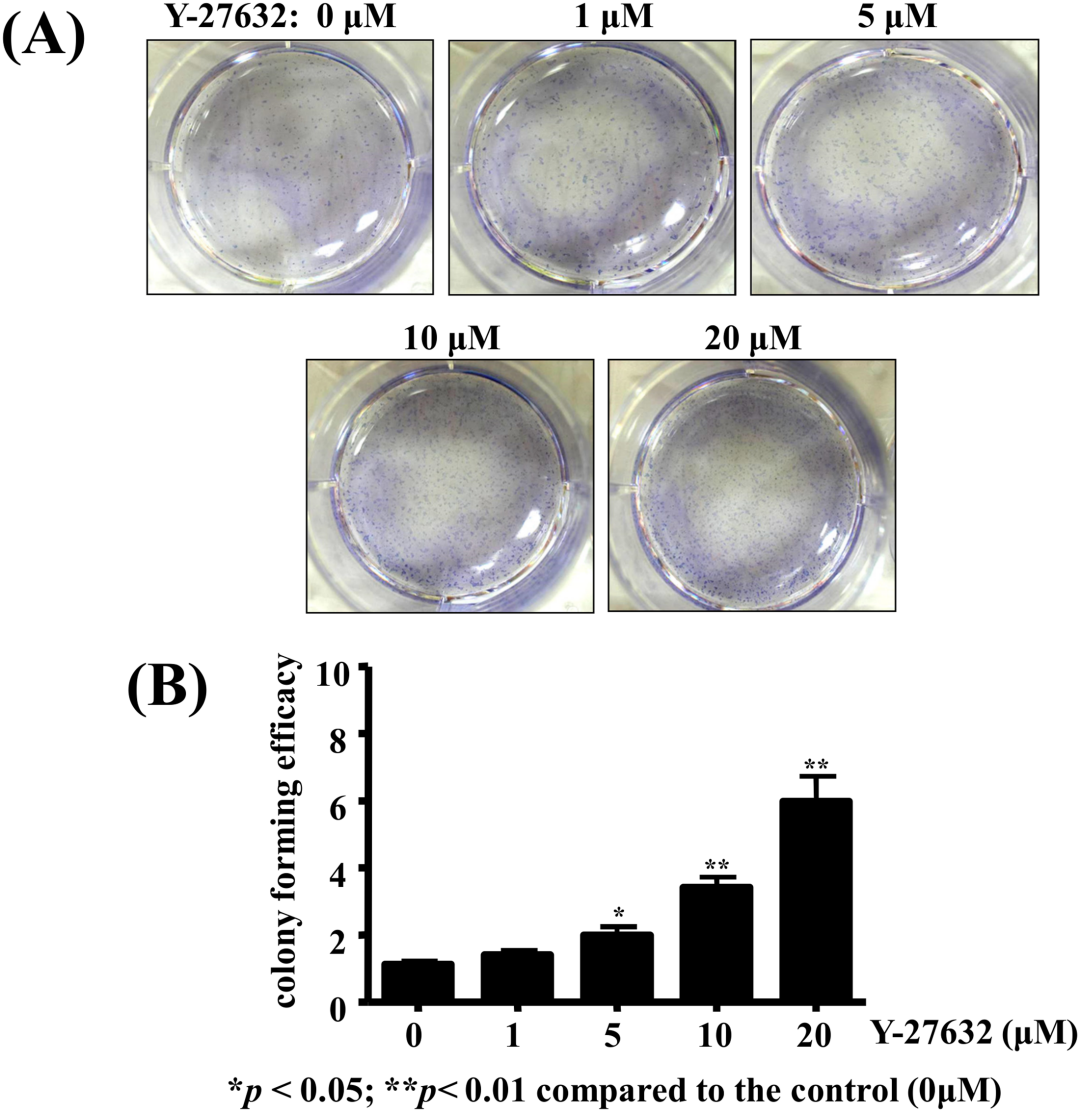
Y-27632 enhanced the colony forming efficiency of human limbal epithelial cells. Human limbal epithelial cells at low cell density were expanded on plastic dishes with or without adding various concentrations of Y-27632 in culture medium for one week. (A) Photographs were taken after fixation and nuclear hematoxylin staining. (B) Numbers of hematoxylin-stained nuclei were counted and statistical analyses were performed using a paired test, and *p<0.05 was considered significant. There was a positive dose-dependent correlation between the concentrations of Y-27632 and colony forming efficiency of human limbal epithelial cells.

The effect of Y-27632 on the ability of cultured limbal epithelial cells to form colonies was evaluated by the colony forming efficiency (CFE) assay. As demonstrated in Fig 3A, one week after the seeding on plastic dishes, multiple colonies with various sizes were formed by human limbal epithelial cells and visibly detected by staining the nuclei with hemotaxylin. The CFE of the cultured limbal epithelial cells was significantly higher in Y37632-treated cultures as compared with the control in a dose-dependent manner (p<0.05 at 5μM; p<0.01 at 10 and 20μM, respectively, Fig 3B).

### Y-27632 increased the expression of Ki67, a proliferation marker, in human limbal epithelial cells

**Fig 4:**
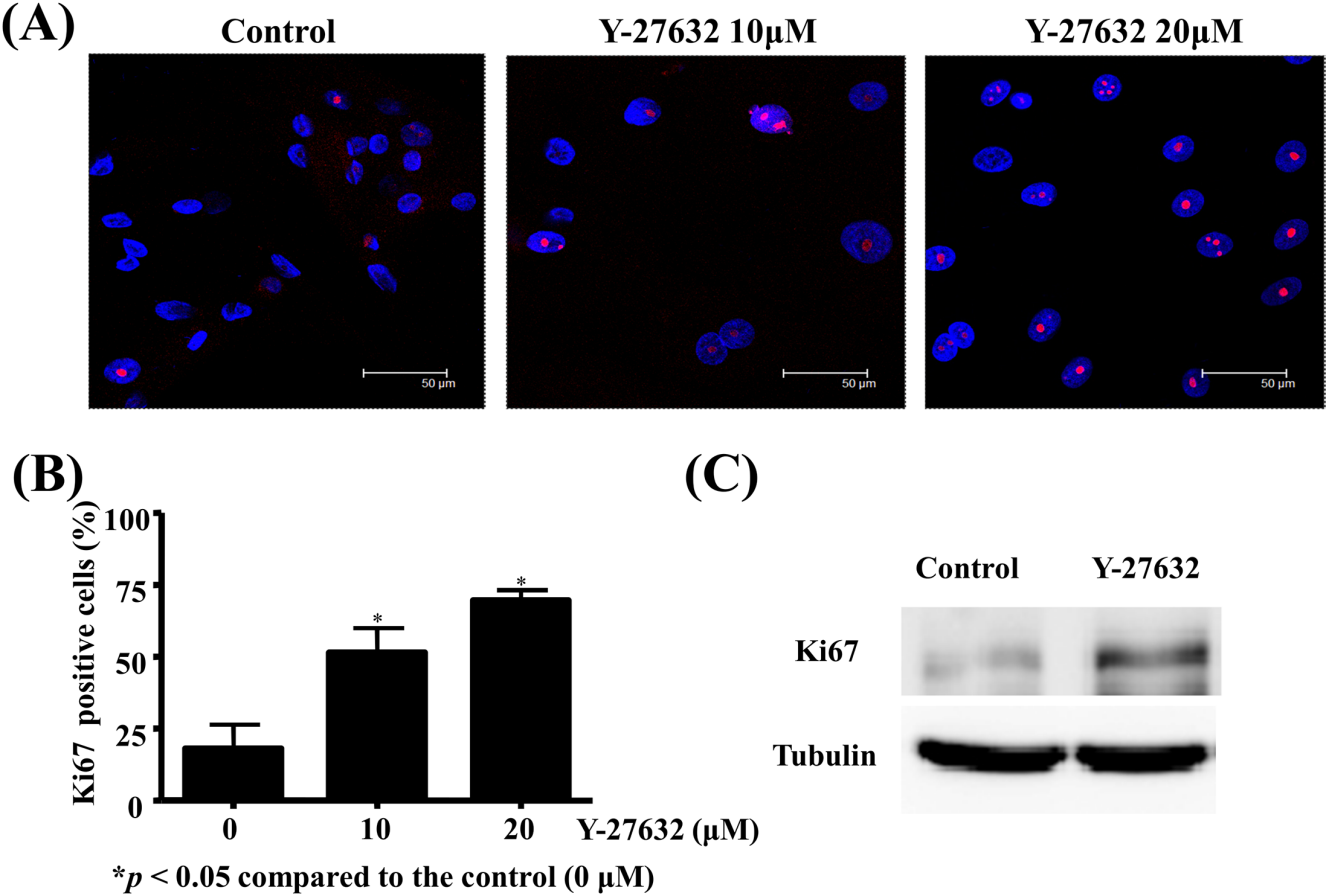
Y-27632 increased expression of Ki67, a proliferation marker, in human limbal epithelial cells. Human limbal epithelial cells were expanded on cover slides in plastic dishes with or without various concentrations of Y-27632 in culture medium for 30 hours. (A) After fixation, immunofluorescent staining was performed with anti-ki67 antibody. The nuclei were counterstained by DAPI (blue). Scale bar, 50 μm. Magnification: 630×. (B) The percentage of Ki67-positive limbal epithelial cells (pink fluorescence in nuclei) under at least 5 high power fields in each slide was counted and statistical analyses were performed using a paired test, and *p<0.05 was considered significant. There was a dose-dependent correlation between the concentrations of Y-27632 and percentage of ki67-positive limbal epithelial cells. (C) Whole cell protein extracts were analyzed by western blot using anti-ki67 antibody with tubulin as an internal control.

It is evident that Y-27632 may induce proliferation of expanded limbal epithelial cells in vitro from the above results. To identify whether Y-27632 is strictly associated with cell proliferation on the molecular level, we used Ki67 as a marker to determine the growth fraction of the limbal epithelial cell population. As shown in Fig 4, nuclear protein Ki67 was positively stained in certain limbal epithelial cells (pink, 4A) and there was a dose-dependent correlation between the concentrations of Y-27632 and percentage of Ki-67 positive limbal epithelial cells (p<0.05 compared to control, 4B). The protein expression level of Ki67 was confirmed to be increased in Y-27632-treated cells by western blot analysis (Fig 4C).

### Y-27632 induced the expression of p63, a presumed limbal stem cell marker, in human limbal epithelial cells

**Fig 5:**
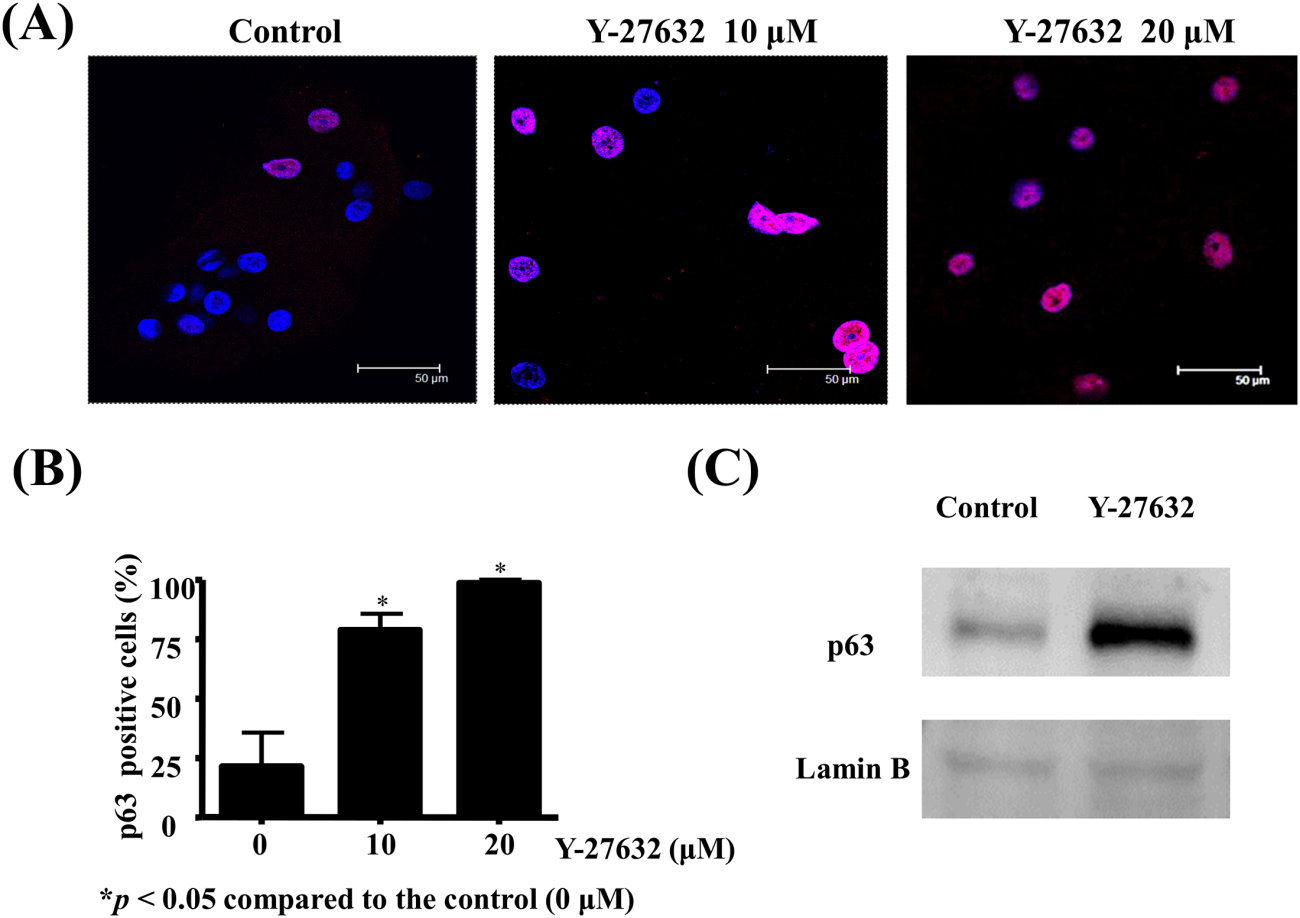
Y-27632 increased expression of p63, a presumed limbal stem cell marker, in human limbal epithelial cells. Human limbal epithelial cells were expanded on cover slides in plastic dishes with or without various concentrations of Y-27632 in culture medium for 30 hours. (A) After fixation, immunofluorescent staining was performed with anti-p63 antibody. The nuclei were counterstained by DAPI (blue). Scale bar, 50 μm. Magnification: 630×. (B) The percentage of p63-positive limbal epithelial cells (pink fluorescence in nuclei) under at least 5 high power fields in each slide was counted and statistical analyses were performed using a paired test, and *p<0.05 was considered significant. There was a dose-dependent correlation between the concentrations of Y-27632 and percentage of p63-positive limbal epithelial cells. (C) Nuclear protein extracts were analyzed by western blot using anti-p63 antibody with lamin B as an internal control.

Transcription factor p63, preferentially expressed in the basal layer of the limbus but not in the corneal epithelium of human corneas, was proposed to be a limbal stem cell marker [17]. To elucidate whether Y-27632-induced proliferation is associated with limbal stem cell proliferation, we performed immunofluorescent staining with p63 antibody on in vitro cultured limbal epithelial cells. Result demonstrated that Y-27632 increased the p63 protein expression in limbal epithelial cells (pink, Fig 5A). The number of p63 positive limbal epithelial cells was quantitatively higher in Y-27632-treated cultures compared to the control and there was a dose-dependent correlation between the concentrations of Y-27632 and percentage of positive staining cells (p<0.05 compared to control, 5B). The protein expression level of p63 was confirmed to be increased in Y-27632-treated cells by western blot analysis (Fig 5C).

### Effects of Y-27632 on the cell cycle distribution and apoptosis of human limbal epithelial cells

**Fig 6:**
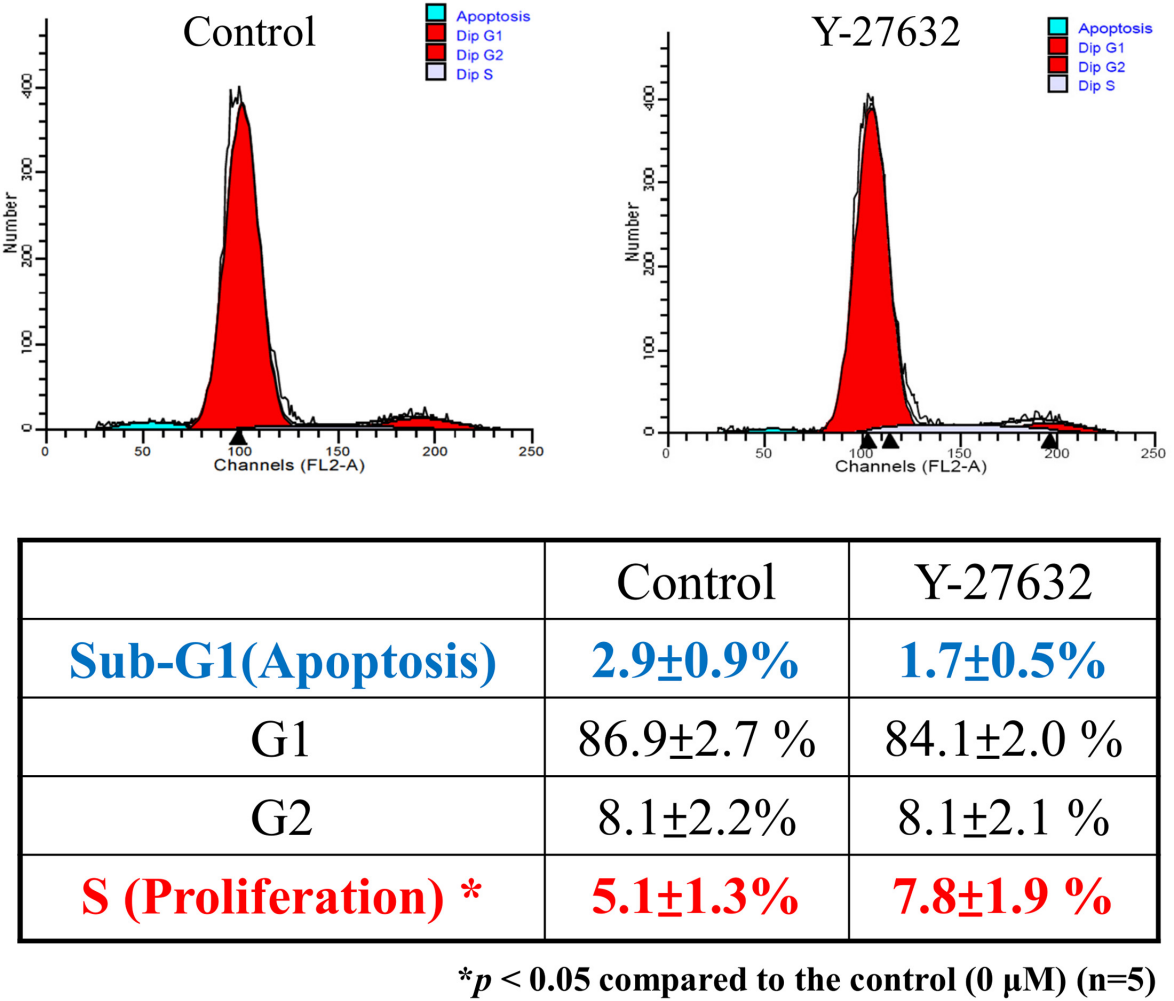
Effects of Y-27632 on the cell cycle distribution and apoptosis of human limbal epithelial cells. Human limbal epithelial cells were expanded on plastic dishes with or without 20uM Y-27632 in culture medium at 37°C for 30 hours. After trypsinization, cells were fixed, stained with PI and analyzed by flow cytometry. Statistical analyses were performed using a paired test, and *p<0.05 was considered significant. The percentage of apoptotic cells (subG1 phase) was reduced from 2.9±0.9% (control) to 1.7±0.5% in the presence of Y-27632 (p = 0.24, n = 5). On the other hand, the percentage of proliferating cells (S-phase) was increased from 5.1±1.3% (control) to 7.8±1.9% in the presence of Y-27632 (p = 0.0295, n = 5).

It has been reported that ROCK inhibitor, Y-27632, was able to prevent apoptosis of dissociated human embryonic stem cells [18]. Therefore, we utilized flow cytometry to investigate whether Y-27632 exerts similar effect on apoptosis of human limbal epithelial cells. The effect of Y-27632 on the cell cycle distribution was shown in Fig 6, demonstrating an increased proportion of limbal epithelial cells in the S phase (p = 0.0295, n = 5). The result was consistent with Fig 2, indicating that Y-27632 is capable of inducing limbal epithelial proliferation. However, despite mild decrease in the number of sub-G1 apoptotic cells in Y-27632-treated group, the statistics was not significantly differed as compared to the sham control (p = 0.24, n = 5).

### Effects of Y-27632 on the expression of Keratin 12, a corneal epithelial differentiation marker, in limbal epithelial cells

**Fig 7:**
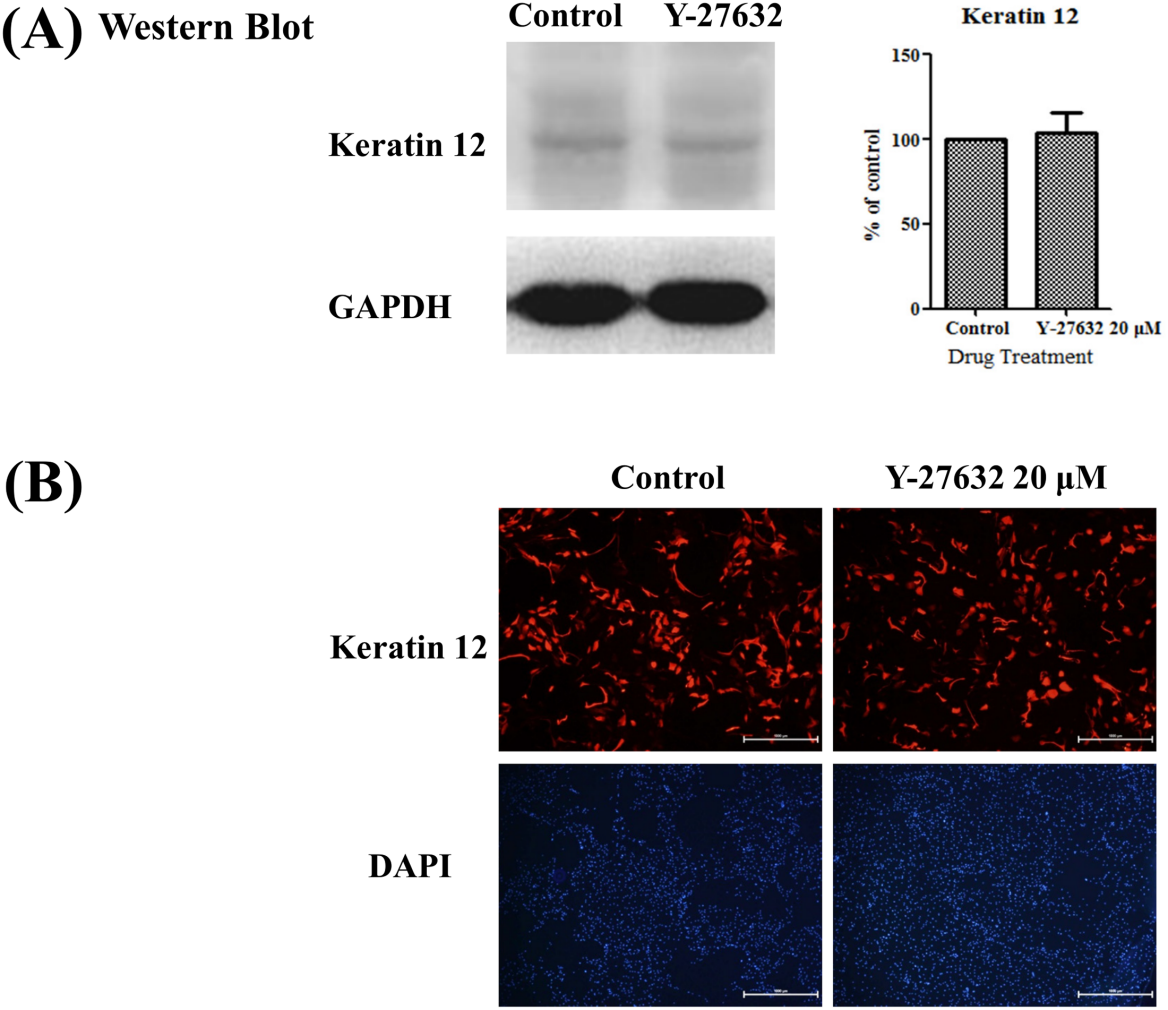
Effects of Y-27632 on the expression of Keratin 12, a corneal epithelial differentiation marker, in limbal epithelial cells. Human limbal epithelial cells were expanded on cover slides in plastic dishes with or without 20μm Y-27632 in culture medium for 30 hours. (A) Total protein extracts were analyzed by western blot using anti-keratin 12 antibody with GAPDH as an internal control. (B) After fixation, immunofluorescent staining was performed with anti-keratin 12 antibody. The nuclei were counterstained by DAPI (blue). There was no significant difference in the expression of keratin 12 between Y-27632 and control groups. Statistical analyses were performed using a paired test, and *p<0.05 was considered significant. Scale bar, 1000 μm. Magnification: 40×.

Since differentiation and proliferation usually go hand in hand, it would be helpful to see how differentiation has been affected by Y-27632. Therefore, we performed both western blot and immunofluorescent staining of a cornea-specific marker, keratin 12, on in vitro cultured limbal epithelial cells. As shown in Fig 7A, keratin 12 protein expression was not significantly differed between the groups (p = 0.764, n = 5). Similarly, keratin 12-positive limbal epithelial cells was evenly distributed between Y-27632-treated and control groups (Fig 7B). Our results indicated that Y-27632 did not affect corneal epithelial cell differentiation.

### Y-27632 promoted in vivo corneal epithelial wound healing

**Fig 8:**
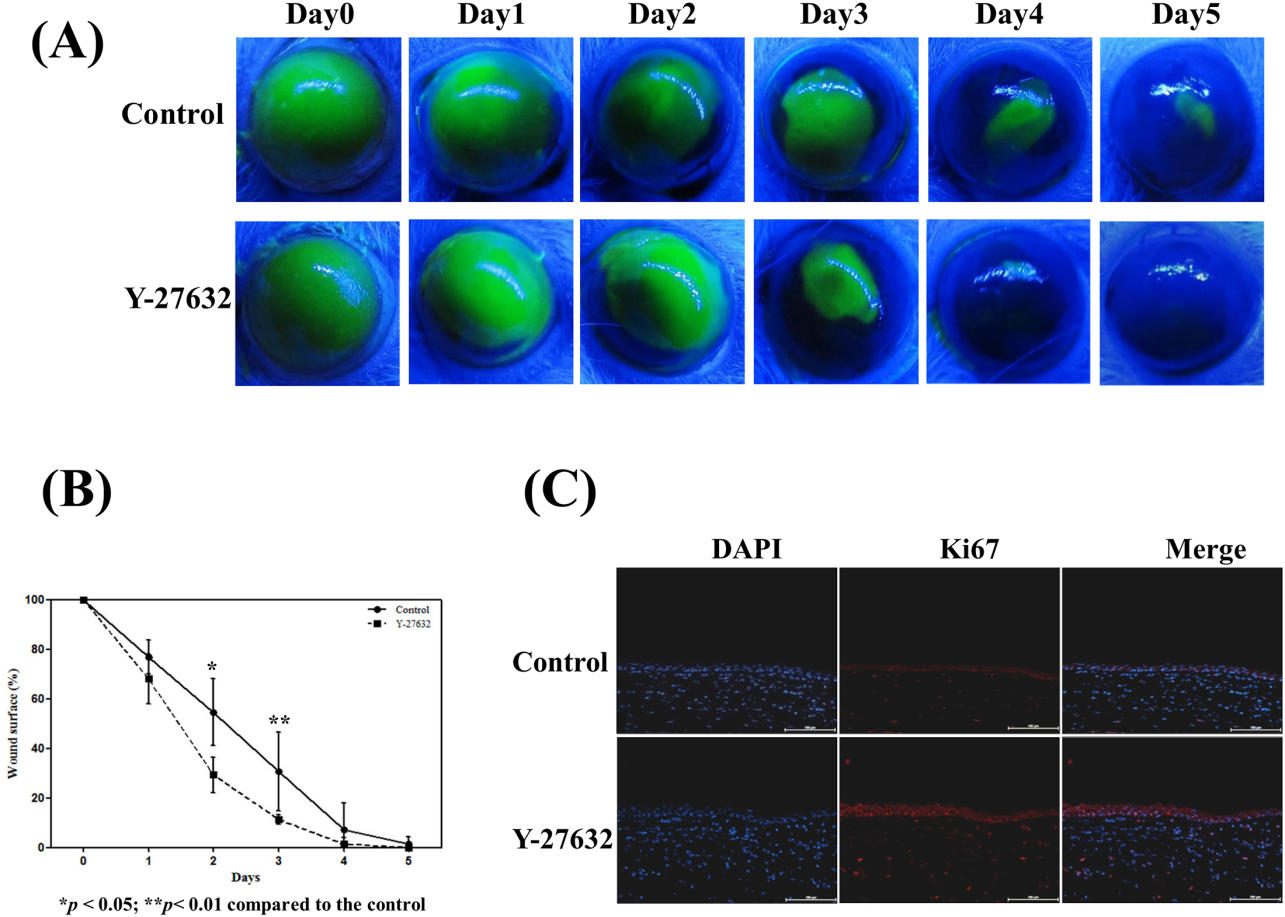
Y-27632 promoted in vivo corneal epithelial wound healing. Corneal epithelial wounds were created in both eyes of Sprague Dawley rats. Y-27632 or PBS was applied topically five times a day to the left or right eye, respectively. (A) After wounding, the area of epithelial defect in each cornea was revealed by staining with 1% fluorescein, and photographed every 24 hours until healed. Representative photographs of corneal healing in five different experiemnets were shown here. The corneal epithelial wounds were rapidly healed in Y-27632-treated eyes. (B) The area of the photographed corneal epithelial defect was measured by a computer-assisted digitizer and statistical analyses were performed using a paired test, and *p<0.05 was considered significant (n = 5, p = 0.02 and 0.002 at day 2 and day 3, respectively). (C). Healed corneas were fixed and stained with anti-Ki67 antibody. Ki67 was more abundantly expressed in the Y-27632-treated corneas, confirming that proliferation was actually increased by Y-27632. The nuclei were counterstained by DAPI (blue). Scale bar, 100 μm. Magnification: 200×.

To investigate the role of ROCK inhibitor in corneal epithelial wound healing, we assessed the effect of Y-27632 on closure rate of epithelial corneal wounding in a rat model. Mechanical corneal wounding was made by a corneal rush ring remover and the remaining epithelial defects were photographed every 24 hours till healing was complete. As shown in Fig 8A, the corneal epithelial wounds in Y-27632-treated group were almost completed by day 4 as compared with the control. Quantitative comparison of the remaining defect area between control and Y-27632 group (Fig 8B) further confirmed the promoting effect of Y-27632 on corneal wound healing (n = 5, p = 0.02 and 0.002 at day 2 and day 3, respectively). To verify whether Ki67 is actually induced in vivo by Y-27632, we performed immunohistochemical staining on completely healed corneas in both groups. We found that Ki67 was more abundantly expressed in the Y-27632-treated corneas (Fig 8C), confirming that proliferation was actually increased by Y-27632.

## Discussion

Maintaining a clear cornea is a prerequisite for excellent vision. Proliferation of limbal epithelial stem cells is the first step to resurface the corneal epithelium in response to any ocular surface insults. Some tuft clinical scenarios such as severe dry eye, limbal stem cell deficiency, diabetes mellitus, and neurotrophic keratitis may lead to persistent corneal epithelial defects [19] which may, when healed, result in corneal scar and opacity. Therefore, it is utmost important to find an efficient treatment to promote corneal reepithelialization. In this study, we evaluated the role of a specific ROCK inhibitor, Y-27632, on in vitro limbal epithelial cell proliferation and in vivo wound healing. We found that Y-27632 induced proliferation of cultivated human limbal epithelial cells. Colony forming efficiency was also enhanced by Y-27632. However, apoptotic limbal epithelial cells were not significantly reduced in Y-27632-treated culture. Moreover, Y-27632 increased the expression of presumed limbal stem cell marker, p63. Finally, by using an in vivo animal model, we demonstrated that application of Y-27632 may promote epithelial wound healing. Our data indicate that ROCK inhibitor, Y-27632, play an important role in limbal epithelial proliferation and corneal wound healing.

The Rho family, including RhoA, RhoB, RhoC, Rac, and CDC42, are small G-proteins. They are active when bind to guanosine triphosphate (GTP), and inactive when bind to guanosine diphosphate (GDP). ROCK 1 and ROCK 2 are among the effectors of RhoA that have been most extensively characterized [20–22]. Once activated, RhoA/ROCK induces myosin light chain (MLC) phosphorylation by inhibiting MLC phosphatase activity, thus increases myosin ATPase activity, leading to the assembly of actin-myosin filaments [23, 24]. In addition to regulating focal adhesions and stress fiber formation, ROCK also plays an essential role in cell migrations, cell cycle control and neurite outgrowth [25]. For instance, increased ROCK activity has been associated with cancer metastasis and tumor cell proliferation [26–28]. In addition, ROCK inhibition with Y-27632, has been shown to inhibit proliferation of several different cell types in vitro [29].

Nevertheless, our in vitro study shows that Y-27632 induces limbal epithelial cell proliferation in a dose-dependent manner (Fig 2). This result is also evidenced by a dose-dependent increase of Ki67 positive cultivated limbal epithelial cells treated with Y-27632 (Fig 4). A recent study indicated that Y-27632 accelerated epithelial wound closure in cultured SV40-immortalized human corneal epithelial cells, but it did not affect basal cell proliferation near a scratch wound [30]. Their studies are in agreement, in part, with ours in that Y-27632 may promote epithelial wound healing. However, our work demonstrated that 20 μm Y-27632 increases 38% human limbal epithelial cell proliferation as compared with the control (Fig 2B). A possible explanation might be that the primary human limbal cultures were used in our study, while they used transformed corneal epithelial cell line. Limbal stem cells are slow cycling cells and possess a higher proliferative capacity [31] compared with the central corneal epithelial cells. Several lines of evidence have also demonstrated that Y-27632 did stimulate proliferation in various cells [14, 32, 33], indicating that Rho/ROCK signaling may have different biological effects on different cell types. The mechanisms by which ROCK inhibitor might induce limbal epithelial cell proliferation remain elusive. In corneal endothelial cells, both cyclin D and cell cycle inhibitor p27kip are involved in Y-27632 induced cell proliferation via phosphoinositide 3-kinase (PI3-K) signaling, a major survival pathway [34]. Specific inactivation of ROCK also increases Ki67 expression and decreased the expression of the cell cycle inhibitors p27kip and p16INK4a on mouse ciliary epithelium, a potential source of stem cells in the adult retina [33]. Taken together, this implies that alteration of the levels of cell cycle regulatory proteins might be involved in the downstream signaling. However, whether the same mechanism is utilized by limbal epithelial cells treated with Y-27632 certainly deserves further investigation.

The present study also demonstrated that human limbal epithelial cells treated with Y-27632 exhibited improved colony-forming efficiency in a dose-dependent manner by enhancing the expansion of the progenitor cells (Fig 3A). We showed that treating limbal epithelial cells with 20 μM Y-27632 increased their cloning efficiency by 6-fold in an in vitro colony forming assay (Fig 3B). Accumulating evidence has also demonstrated that Y-27632 greatly increased the cloning efficiency of embryonic or adult stem cells, including limbal epithelial cells [35–37]. Moreover, human embryonic stem cells treated with 20 μM Y-27632 maintained a high level expression of pluripotency-related genes [38]. The mechanism by which Y-27632 promotes limbal epithelial cells colony forming efficiency remains unclear. Previous reports indicated that Y-27632 may improve the rapid adherence of limbal epithelial cells in the initial inoculation [37]. The fact that Y-27632 may enhance the expansion of limbal epithelial cells is very important in some aspects in terms of clinical transplantation. First, the current mainstream of limbal stem cell therapy for reconstructing the ocular surface depends greatly on the number and survival of the transplanted limbal stem cells [39]. Therefore, if we could obtain an adequate number of limbal progenitor cells in vitro with small molecules such as ROCK inhibitor, we may improve the successful rate of LSC transplantation using ex vivo cultivation strategy. Second, the possibility of xenocontamination during transplantation by mouse feeder layer, fetal bovine serum, or recombinant growth factors [40] could therefore be avoided.

We also confirmed that Y-27632 increased the expression of the putative limbal stem cell markers P63 (Fig 5) in a dose-dependent manner, suggesting that Y-27632 might be implicated in the inhibition of differentiation of limbal epithelial cells in vitro. However, our results indicated that Y-27632 did not alter the expression of keratin 12 on in vitro cultured limbal epithelial cells (Fig 7). In fact, previous study has demonstrated that ROCK 1, the downstream effector of RhoA, is preferentially expressed in the corneal epithelial cells than in the limbal counterpart [41], suggesting that increased expression of ROCK 1 was one of the changes associated with limbal to corneal epithelial differentiation [41]. On the other hand, human pluripotent stem cells may retain typical morphology, stable karyotype, and expression of pluripotency markers even after a substantial number of passages under Y-27632 treatment [18]. It has also been evidenced that Rho GTPase modulation (inactivation or activation) may have different effects on the expression of retinal progenitor genes and proliferation in the adult ciliary epithelial progenitor cells of rodent eyes [33]. Their study indicated that activation of Rho GTPases increased progenitor profile in ciliary epithelial cells, observed by coexpression of Pax6 and Chx10 [33]. However, the current study demonstrated that ROCK inhibition induces LEC proliferation, up-regulates Ki67 and P63 expression in human LECs. Therefore, further study is mandatory to verify whether Y-27632 is capable of maintaining limbal progenitor cell stemness.

ROCK-mediated actin-myosin contractile force plays an important role in regulating apoptosis [42]. The inhibition of ROCK activity has been reported to have pro-survival effects in various cell type including human embryonic stem cells [43], astrocytes [44] and corneal endothelium [14]. Moreover, the addition of Y-27632 also increased Oct-3/4 expression in small colonies of human embryonic stem cells [45]. In this study, the apoptotic cells in sub G1 phase of cultivated human LECs treated by Y-27632 were only mildly decreased and did not reach statistical significance as compared with the control (Fig 6). But we did showed that about 1.6 folds of LECs entered the synthetic (S) phase in Y-27632- treated cultures, which was compatible with the cell proliferation assay. If the ROCK inhibitor significantly inhibits apoptosis of limbal epithelial cells, it may facilitate LEC expansion *in vitro* and would be beneficial for ocular surface reconstruction. However, further study is necessary to investigate whether inhibition of ROCK activity would be compatible with the survival of differentiated cells, since ROCK inhibitor may actually induce apoptosis in corneal epithelial cells [46].

Recent study demonstrated that RhoA is involved in regulating corneal epithelial cell migration and focal adhesion formation through ROCK and its inhibition with Y-27632 promotes basal and growth factor—enhanced human corneal epithelial cell migration [30]. Furthermore, topical Y-27632 eye drops have beened for the treatment of corneal endothelium disorders [16]. Our study also demonstrated that topical Y-27632 application accelerates corneal epithelial wound closure and shortens the wound healing time (Fig 8). Since ROCK inhibitors such as Y-27632 and Fasudil are already used clinically [23, 24], this would indicate that they are safe for use in ocular surface diseases. Under certain clinical scenarios with delayed corneal epithelial wound healing such as diabetic keratopathy, post-vitrectomy persistent epithelial defects and partial LSCD, ROCK inhibitors may be an useful therapeutic to promote wound closure, which is very important for reducing the risk of infectious keratitis and preventing poor visual outcomes.

In summary, the present study demonstrated that Y-27632 might induce proliferation of *in vitro* cultured limbal epithelial cells and promote *in vivo* epithelial wound healing, which open a new therapeutic strategy for LSCD. However, further study is mandatory to investigate the underlying molecular mechanisms.
